# A case of lymphomatosis cerebri mimicking inflammatory diseases

**DOI:** 10.1186/s12883-016-0655-7

**Published:** 2016-08-08

**Authors:** Takenobu Murakami, Kenji Yoshida, Mari Segawa, Akioh Yoshihara, Akihiko Hoshi, Koichiro Nakamura, Masahiro Ichikawa, Osamu Suzuki, Yuichi Yokoyama, Yasuko Toyoshima, Yoshihiro Sugiura, Hiroshi Ito, Kiyoshi Saito, Yuko Hashimoto, Akiyoshi Kakita, Hitoshi Takahashi, Yoshikazu Ugawa

**Affiliations:** 1 Department of Neurology, Fukushima Medical University, 1, Hikarigaoka, Fukushima, 960-1295 Japan; 2 Advanced Clinical Research Center, Fukushima Global Medical Science Center, Fukushima Medical University, 1, Hikarigaoka, Fukushima, 960-1295 Japan; 3 Department of Neurology, Fukushima Red Cross Hospital, 11-31, Iriecho, Fukushima, 960-8530 Japan; 4 Department of Neurosurgery, Fukushima Medical University, 1, Hikarigaoka, Fukushima, 960-1295 Japan; 5 Department of Pathology, Fukushima Medical University, 1, Hikarigaoka, Fukushima, 960-1295 Japan; 6 Department of Pathology, Brain Research Institute, Niigata University, 1-757, Asahimachi-dori, Chuo-ku, Niigata, 951-8585 Japan

**Keywords:** Lymphomatosis cerebri, Fever, Nonvasculitic autoimmune inflammatory meningoencephalitis, Brain biopsy, Magnetic resonance imaging

## Abstract

**Background:**

Lymphomatosis cerebri (LC) is a rare subtype of primary central nervous system malignant lymphoma. The typical features of this disease exhibited on magnetic resonance imaging (MRI) without contrast enhancement are similar to those observed with diffuse leukoencephalopathy, mimicking white matter disorders such as encephalitis. Clinical features and examination findings that are suggestive of inflammatory diseases may indeed confound the diagnosis of LC.

**Case presentation:**

A 66-year-old woman with continuous fever over a two-month period developed left hemiparesis despite presenting in an alert state with normal cognitive function. Sampling tests showed autoantibodies in the serum and inflammatory changes in the cerebrospinal fluid. The results from an MRI demonstrated multiple non-enhanced brain lesions in the splenium of the corpus callosum and deep white matter. Single photon emission computed tomography revealed increases in blood flow in the basal ganglia, thalamus and brainstem. No systemic malignancies were found. The patient was suspected of having a diagnosis of nonvasculitic autoimmune inflammatory meningoencephalitis and treated with intravenous methylprednisolone pulse therapy. Her fever transiently dropped to within the normal range. However, she had a sudden seizure and a second MRI exhibited infiltrative lesions gradually extending throughout the whole brain. We performed a brain biopsy, and LC was histologically diagnosed. The patient received whole-brain radiation therapy, which diminished the fever and seizures. The patient died one year after the initial onset of fever.

**Conclusions:**

The present case yields an important consideration that brain neoplasms, especially LC, cannot be ruled out, even in cases with clinical characteristics and examinations consistent with inflammatory diseases. Careful follow-up and histological study are vital for the correct diagnosis of LC.

## Background

Primary central nervous system malignant lymphoma (PCNSL) generally forms either a single or multiple isolated, solitary mass lesions in the brain parenchyma. A very rare PCNSL variant, known as lymphomatosis cerebri (LC), presents as the diffuse infiltration of lymphoma cells in gray and white matter without the formation of a cohesive tumor mass [1, 2]. In this study, we report the case of a patient with continuous fever and multiple brain lesions. Because the patient had autoantibodies with no positive findings of malignancy, she was initially suspected of having a diagnosis of nonvasculitic autoimmune inflammatory meningoencephalitis (NAIM). Because extensive immunotherapy failed to result in symptomatic improvements, we performed a brain biopsy, which revealed diffuse infiltration of lymphoma cells in the brain parenchyma. Thus, a diagnosis of LC was made. We showed that LC could follow a clinical course mimicking inflammatory disease and that careful attention was required to distinguish LC from inflammatory diseases.

## Case presentation

A 66-year-old woman, who had been healthy with no relevant previous medical history, complained of continuous fever starting two months before initial presentation. She realized weakness on her left extremities and was admitted to a city hospital for evaluation of fever and left hemiparesis. Then, she was transferred to our hospital for further evaluation three weeks later. On examination, her general condition was normal, except for an elevated body temperature (38.0° Celsius). No lymph-nodes were palpable. Additionally, she did not complain of Sicca symptoms, night sweats or weight loss. The patient was alert and had normal cognitive function. She had left hemiparesis (grade 4 as measured by the Manual Muscle Strength Testing) with bilateral brisk deep tendon reflexes and extensor plantar responses. Sensory extinction was noted in her left extremities.

The blood sample tests showed a slightly elevated C-reactive protein level (0.68 mg/dl) and erythrocyte sedimentation rate (17 mm/h) as well as a high titer of antinuclear antibodies (x1280, speckled pattern). Anti-SS-A and anti-Ro52 antibodies were positive but anti-SS-B antibody was negative. Lip biopsy showed no inflammation. Immunoblotting revealed IgG-k monoclonal gammopathy; however, bone marrow histology showed 10 % proliferation of clonal plasma cells without light chain restriction, and Bence-Jones protein was not found. Tumor markers were negative and soluble interleukin-2 receptor (sIL-2R) was within the normal range. Cerebrospinal fluid (CSF) showed slight pleocytosis (11/μl; mature lymphocyte 94 %, no atypical cells). However, normal ranges of glucose and protein levels (73 mg/dl and 26 mg/dl, respectively) were observed. We also observed increments of both the IgG index (1.26) and interleukin 6 (29.4 pg/ml) but no increment of sIL-2R.

**Fig. 1:**
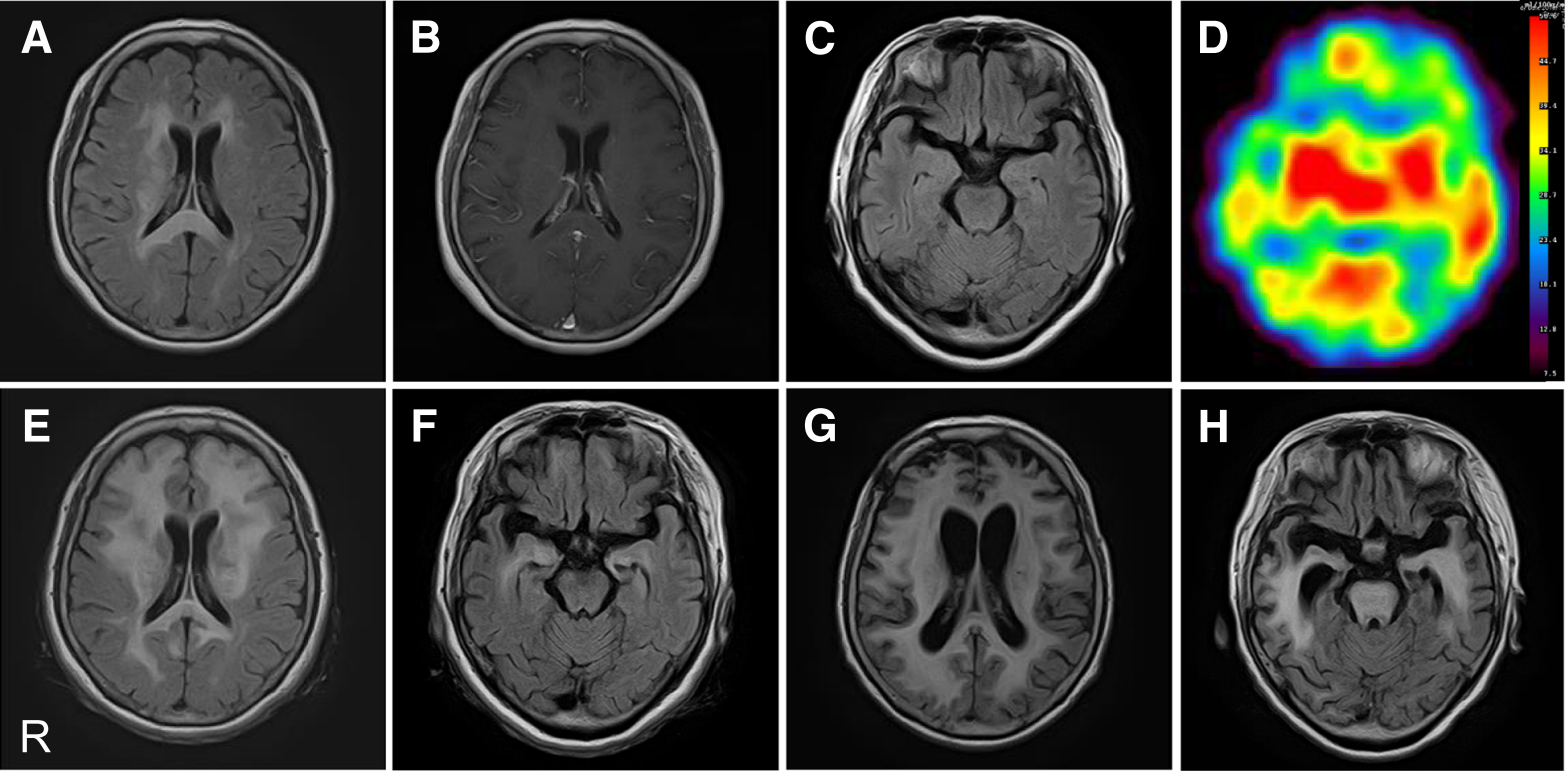
Brain magnetic resonance images and single photon emission computed tomography (SPECT). **a**-**c** Two months after fever onset, fluid-attenuated inversion recovery (FLAIR) images show diffuse high signal intensity in the splenium of the corpus callosum and right-hemisphere dominant subcortical white matters. However, this finding is (**a**) neither exhibited in the temporal lobes nor exhibited in the brainstem (**c**). No contrast enhanced lesions are observed in gadolinium-enhanced T1-weighted images (**b**). **d** SPECT study shows hyperperfusion in the basal ganglia, thalamus and brainstem. (E-H) Axial FLAIR images. Approximately five months after the onset of fever, hyperintensity lesions extended to the frontotemporal lobes, bilaterally (**e**, **f**). Twelve months after the onset of fever, FLAIR images show diffuse progression of infiltrative lesions in the whole brain including the brainstem with systemic brain atrophy (**g**, **h**)

Brain magnetic resonance imaging (MRI) exhibited hyperintense signals in the splenium of the corpus callosum and right-hemisphere dominant deep subcortical white matter without contrast enhancements (Fig. 1a-c). The brain lesions showed normal concentration patterns of choline, creatine and N-acetyl aspartate using MR spectroscopy. However, ^123^I-IMP single photon emission computed tomography (SPECT) demonstrated blood flow increases in the basal ganglia, thalamus and brainstem (Fig. 1d), and ^201^Tl-scintigraphy revealed no abnormal accumulation in any part of the brain. ^67^Ga-scintigraphy found no evidence of systemic malignancy.

We tentatively diagnosed the patient as having NAIM associated with anti-SS-A and anti-Ro52 antibodies, as well as monoclonal gammopathy of undetermined significance. She was treated with intravenous methylprednisolone (mPSL) pulse therapy (1 g during the course of three days) and her fever transiently ceased. Three months after the onset of fever, she suddenly developed generalized tonic-clonic seizures (GTCS), which remained uncontrolled after the administration of antiepileptic medications. Intravenous immunoglobulin (IVIG) (0.4 g/kg in five days) had no beneficial effects. A second brain MRI showed extensive lesions that had developed in the bilateral frontotemporal lobes (Fig. 1e, f).

**Fig. 2:**
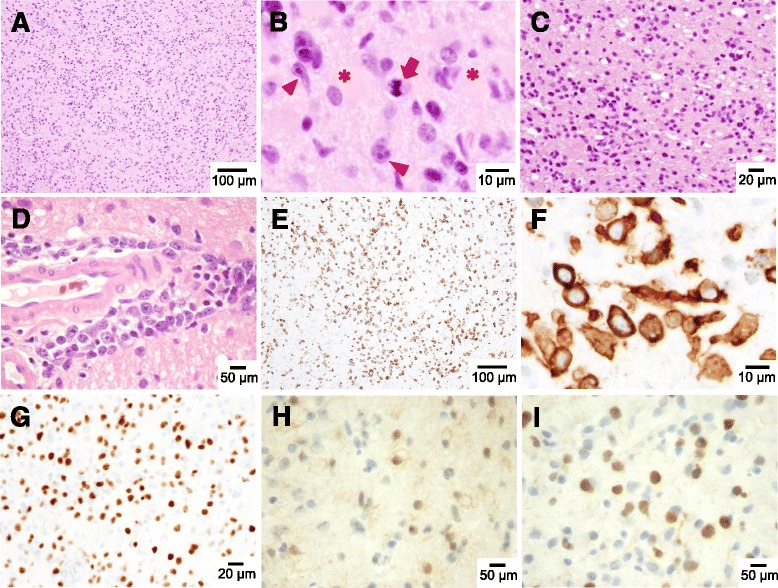
Pathological findings of brain biopsy. **a**-**c** Hematoxylin-eosin staining revealed diffuse infiltration of atypical cells in cortical gray (**a**, **b**) and deep white matter (**c**). Lymphoma cells show enlarged round nuclei, thin cytoplasmic rims and occasional nucleoli (arrowheads). Mitotic cells (*arrow*) and reactive astrocytes with eosinophilic cytoplasm (*asterisk*) are identified (**b**). Perivascular infiltration of the atypical cells was partly observed (**d**). Immunohistochemical studies revealed the infiltration of CD20 positive cells in the parenchyma (**e**, **f**) and numerous MIB-1 positive cells (**g**). BCL-6 and MUM-1 immnunostainings highlight a non-germinal center B-cell pattern (**h**, **i**)

Brain specimens were sampled from the right frontal lobe using a brain biopsy. The histopathological examination showed atypical lymphoid cells with enlarged round nuclei diffusely infiltrating the cortical gray and subcortical white matters. These atypical cells were not cohesive, nor did they form a mass lesion. Perivascular tumor-cell cuffing was partly observed but not conspicuous. No necrosis was present, and mitotic figures were scant in number. Atypical cells were strongly labeled by antibodies for CD20, a B-cell marker. Additional immnunohistochemical studies revealed that tissue was positive for BCL-2, BCL-6 and MUM-1 but negative for CD5 and CD10 (Fig. 2a-i). A diagnosis of LC variant of diffuse large B-cell lymphoma, non-germinal center B-cell pattern was made based on these histopathological and immunohistochemical features.

The patient had repeated GTCS, and accompanying severe aspiration pneumonia. She needed antibiotics and mechanical ventilator support. Considering the patient’s exhausted condition, we and her family chose to perform whole-brain radiation therapy (total 30 Gy) but not high-dose chemotherapy or rituximab therapy because the main adverse effect, bone marrow suppression, might be critical for her prognosis. Fever and seizures ceased for two months during and after the radiation therapy; however, she never regained consciousness. The follow-up MRIs revealed the progression of the lesions throughout the whole brain accompanying diffuse brain atrophy (Fig. 1g, h). She died one year after the initial onset of fever. No autopsy was permitted.

## Conclusions

Unlike nodular lesions with contrast enhancement of PCNSL [3, 4], MRI findings of LC are generally consistent with diffuse leukoencephalopathy with absent or very faint patchy contrast enhancement, mimicking various white matter disorders [1, 2]. Therefore, diagnosing LC is a difficult challenge and cannot be accomplished using conventional MRI alone. Pathological examination is crucial for making an accurate diagnosis. Pathological examination should show diffuse infiltration of lymphoma cells in the parenchyma without resulting tissue destruction. Furthermore, lack of contrast enhancement in MRI and no destroyed vessels in the histopathological study are assumed to reflect an intact blood–brain barrier [1].

Previous reports of LC have shown several clinical features of this disease, such as subacute progressive dementia, personality changes, unsteady gait and epileptic seizures [5–7]: however, to the best of our knowledge, LC in patients with continuous fever has not been reported to date [3, 8].

In the present case, we first suspected NAIM based on findings that were suggestive of an inflammatory disease, such as the existence of autoantibodies with inflammatory CSF changes, multiple non-enhanced brain lesions on MRI and the transient improvement experienced after the initial mPSL treatment. NAIM is characterized as a steroid-responsive encephalopathy associated with nonspecific autoantibodies [9]. In steroid-resistant cases of NAIM, the use of IVIG results in a sufficiently good outcome [10]. In our case, however, the resistance to the subsequent immunotherapy led us to perform the brain biopsy, from which we were able to make a diagnosis of LC. A transient benefit from the mPSL may be explained by the reduction of the lymphocytotoxic effects.

Although there are no detectable lesions observed during the first experimental MRI, hyperperfusion lesions present in the basal ganglia, thalamus and brainstem, which were observed during the SPECT, might reflect infiltration of LC, indicating that the continuous fever may be explained by dysfunction of thermoregulation in the hypothalamus.

The prognosis of LC is generally considered to be poorer than that of PCNSL, and most LC patients are reported as dying within six months of the initial onset [5, 11]. In contrast, some recent LC cases diagnosed at early stages by pathological examination responded favorably to appropriate treatments [12, 13]. Therefore, early pathological diagnosis should be undertaken in patients suspected of having LC.

In patients with atypical presentations exhibiting features of NAIM, neoplastic disorders of the brain, especially LC, should be considered. Careful follow-up and timely tissue diagnosis are important to determine specific treatments and interventions.

## Abbreviations

CSF, cerebrospinal fluid; FLAIR, fluid-attenuated inversion recovery; GTCS, generalized tonic-clonic seizures; IVIG, intravenous immunoglobulin; LC, lymphomatosis cerebri; mPSL, methylprednisolone; MRI, magnetic resonance imaging; NAIM, nonvasculitic autoimmune inflammatory meningoencephalitis; PCNSL, primary central nervous system malignant lymphoma; sIL-2R, soluble interleukin-2 receptor; SPECT, single photon emission computed tomography
